# Onset of Epstein-Barr virus-associated hemophagocytic lymphohistiocytosis triggered by sudden reactivation of asymptomatic latent Epstein-Barr virus infection

**DOI:** 10.1016/j.htct.2025.103939

**Published:** 2025-07-15

**Authors:** Masanari Mizuki, Takuya Terakawa, Yuka Umeki, Hitomi Matsunaga, Yoshiki Matsuoka, Wataru Nakahara, Shogo Matsui, Mako Ikeda, Kodai Ueshima, Yuya Hayasaka, Nayu Matsuoka, Yuma Tada, Takahiro Matsui, Kazumasa Oka, Shuji Ueda

**Affiliations:** a Department of Hematology, Hyogo Prefectural Nishinomiya Hospital, Nishinomiya, Hyogo, Japan; b Department of Hematology, Osaka International Cancer Institute, Osaka, Japan; c Department of Pathology, Osaka University Graduate School of Medicine, Suita, Osaka, Japan; d Department of Pathology, Hyogo Prefectural Nishinomiya Hospital, Nishinomiya, Hyogo, Japan

## Introduction

Hemophagocytic lymphohistiocytosis (HLH) is a life-threatening hyperinflammatory disease characterized by hypercytokinemia and immune-mediated injury of multiple organ systems. HLH is categorized into two subgroups. Primary HLH is an inherited immune disorder, whereas secondary HLH develops in various settings, such as infection, autoimmune disorder, and malignancy.^1^ Epstein-Barr virus (EBV) is the most frequent cause of HLH associated with infection. EBV-associated HLH (EBV-HLH), like HLH due to other causes, is clinically characterized by fever, splenomegaly, and cytopenia with histologic evidence of hemophagocytosis, along with extremely high levels of serum ferritin and soluble interleukin-2 receptor (sIL-2R). EBV-HLH has a high rate of mortality, often resulting from multiple organ failure.^1^^,^^2^ Immunomodulatory treatment, including etoposide, has demonstrated effectiveness in controlling EBV-HLH, although refractory cases require multiagent chemotherapy or hematopoietic stem cell transplantation.^1^

EBV-HLH can occur following primary EBV infection or as a result of EBV reactivation due to EBV-associated diseases, such as chronic active EBV disease (CAEBV) or certain malignancies.^2^ However, it is not clear whether EBV-HLH can be caused by reactivation of asymptomatic latent EBV infections. Here, we report such a case involving a patient who developed EBV-HLH as a result of sudden reactivation of latent EBV infection.

## Case report

A 36-year-old Japanese man who had been healthy all of his life was admitted to our hospital with a one-month history of fever and anorexia. Review of personal and family history revealed no signs of immunodeficiency. A peripheral blood count showed a white blood cell count of 2.0 × 10^9^/L, a hemoglobin level of 13.6 g/dL, and a platelet count of 148 × 10^9^/L. Morphologic analysis of peripheral blood showed 6.5 % atypical lymphocytes. Biochemical studies revealed hepatic dysfunction and significantly elevated levels of serum ferritin and sIL-2R (Table 1).

**Table 1:** Laboratory findings of the patient.

	Days after hospitalization				Reference range
	1	6	8	10	
WBCs (× 10^9^/L)	2.00	1.06	1.31	2.41	3.30–8.60
Neutrophils (× 10^9^/L)	1.290	0.763	0.983	2.085	
Lymphocytes (× 10^9^/L)	0.650	0.170	0.183	0.133	
Hb (g/dL)	13.6	11.2	10.8	7.7	13.7–16.8
Platelets (× 10^9^/L)	148	85	59	50	158–348
T-bil (mg/dL)	0.70	0.91	1.33	0.99	0.40–1.50
AST (U/L)	83	199	506	451	13–30
ALT (U/L)	57	158	554	449	10–42
LDH (U/L)	735	786	908	1,308	124–222
CRP (mg/dL)	1.65	3.34	3.94	7.79	0.00–1.14
Ferritin (ng/mL)	1,583.7	N/A	11,162.5	20,064.4	14.4–303.7
sIL-2R (U/mL)	8,847.0	N/A	N/A	28,114.0	122.0–496.0

WBC: white blood cell; ALT: alanine aminotransferase; AST: aspartate aminotransferase; CRP: C-reactive protein; Hb: hemoglobin; LDH: lactate dehydrogenase; N/A: not available; sIL-2R: soluble interleuikin-2 receptor; T-bil: total bilirubin.

**Figure 1:**
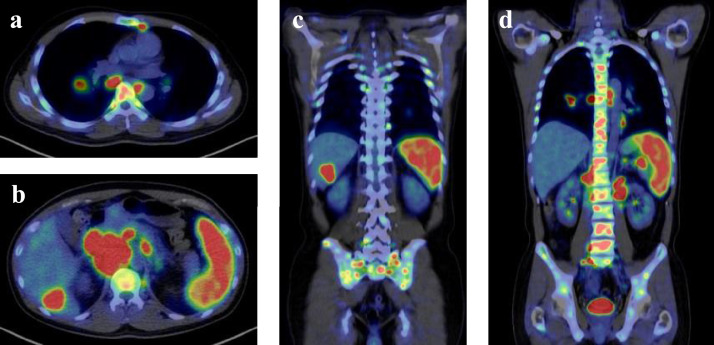
Positron emission tomography-computed tomography demonstrated pathological ^18^F-fluorodeoxyglucose uptake in mediastinal lymph nodes (a, d), abdominal lymph nodes (b, d), spleen (b, c, d), liver mass (b, c), and systemic bone (a, b, c, d).

A contrast-enhanced computed tomography (CT) scan revealed enlarged mediastinal and abdominal lymph nodes, splenomegaly, and a space-occupying lesion in segment 6 of the liver. A positron emission tomography-CT (PET-CT) scan showed pathologic uptake of ^18^F-fluorodeoxyglucose (FDG) in mediastinal and abdominal lymph nodes, spleen, liver lesion, and systemic bone (Figure 1). Examination of the bone marrow aspirate by May-Grünwald-Giemsa staining showed mild hemophagocytosis but no abnormal cells. Flow cytometry analysis of bone marrow cells revealed no neoplastic cell populations, and immunoglobulin heavy chain and T-cell receptor beta chain rearrangement studies detected no rearrangement bands. After admission to our hospital, the patient had a persistent high fever and showed signs of exacerbated pancytopenia and liver dysfunction (Table 1). He met all eight clinical and laboratory diagnostic criteria outlined in the HLH-2004 diagnostic and therapeutic guidelines for HLH and was diagnosed with secondary HLH.

**Figure 2:**
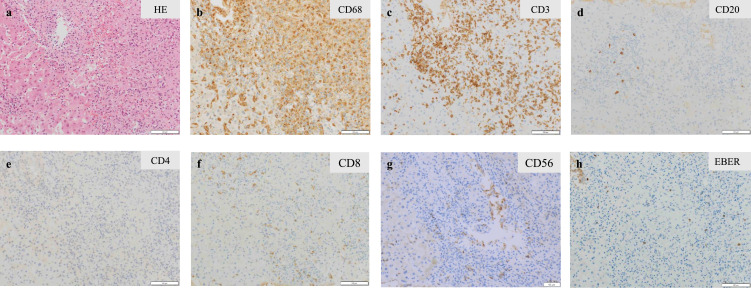
Histopathological findings of liver mass biopsy were consistent with hemophagocytic lymphohistiocytosis. Hematoxylin and eosin stain (a), immunohistochemical stain for CD68 (b), CD3 (c), CD20 (d), CD4 (e), CD8 (f), CD56 (g), and in-situ hybridization for Epstein-Barr virus-encoded early RNA (h). HE: hematoxylin and eosin; EBER: Epstein-Barr virus-encoded early RNA.

A variety of conditions can cause secondary HLH, including infection, autoimmune disorder, and malignancy. There were no clinical or laboratory findings to suggest the presence of autoimmune disease in this patient. We conducted further evaluations based on EBV-related assays. Antibody titers for EBV viral capsid antigen (VCA) IgG, VCA IgM, early antigen (EA) IgG, and nuclear antigen were 1:320, <1:10, 1:10, and 1:80, respectively. Levels of EBV DNA in plasma (4.85 log IU/mL) and in whole blood (5.48 log IU/mL) were significantly elevated. EBV clonality was not detected by terminal repeat analysis of the EBV genome. Percutaneous biopsy of the segment 6 liver mass revealed a proliferation of CD68-positive epithelial histiocytes (i.e., activated macrophages) accompanied by small lymphocytes. Most of the lymphocytes that clustered with these histiocytes were T cells, with a predominance of CD8-positive T cells; EBV-encoded early RNA (EBER)-positive cells were also present (Figure 2). Bone marrow biopsy confirmed nearly the same histology. These pathologic findings were consistent with HLH. On the basis of these results, together with the findings of EBV activation, the patient was diagnosed with EBV-HLH.

His general condition deteriorated rapidly, including impaired consciousness. Levels of serum ferritin (20,064.4 ng/mL) and sIL-2R (28,114.0 U/mL) were markedly elevated (Table 1). Serum interferon-gamma levels, which are considered to play a particularly key role in the cytokine storm in HLH, also were strikingly high at 50 IU/mL (reference range, ≤ 0.1 IU/mL).^1^ We started pulse steroid therapy on admission Day 10, but the treatment was unsuccessful. A reduced dose of SMILE (steroid dexamethasone, methotrexate, ifosfamide, l-asparaginase, and etoposide) chemotherapy was administered as salvage therapy. One week after the start of SMILE therapy, the patient’s overall condition began to improve. After completion of the first course of SMILE therapy, his liver function, sIL-2R level, and ferritin level improved significantly, and his general condition demonstrated a complete recovery. A CT scan of the chest and abdomen revealed significantly decreased sizes of the previously enlarged lymph nodes and liver mass.

Because the reduction of the EBV DNA load in the blood after two cycles of SMILE therapy was insufficient (from 5.48 log IU/mL to 3.48 log IU/mL), the patient underwent allogeneic hematopoietic stem cell transplantation from a human leukocyte antigen–matched unrelated donor. The patient has since maintained a stable condition and achieved an undetectable level of EBV DNA.

## Discussion

We report here a case involving sudden onset of EBV-HLH triggered by reactivation of latent EBV infection. The clinical course of this patient included no features suggesting the presence of any EBV-related disease, such as CAEBV or an associated malignancy, including malignant lymphoma. Serologic testing did not reveal high antibody titers for EBV VCA IgG or EA IgG, which are seen in most cases of CAEBV. Antibody titers for EBV VCA IgG, VCA IgM, and nuclear antigen were 1:320, <1:10, and 1:80, respectively, suggesting latent EBV infection.^3^ Additionally, the positive finding of EA IgG antibodies with a low titer (1:10) suggested reactivation of latent EBV infection.

EBV-HLH is known to occur following primary EBV infection or as a result of reactivation due to EBV-related diseases, such as CAEBV or certain malignancies, and is most common among children and young adults.^2^ It is not clear whether EBV-HLH can result from asymptomatic latent EBV infection in the absence of immunodeficiency. However, the present case is considered to represent EBV-HLH due to a reactivation of latent EBV infection. Some reported cases of adult-onset EBV-HLH include previously healthy individuals whose condition is unlikely to have resulted from primary EBV infection, CAEBV, or EBV-related malignancies.^4^^,^^5^ There is a report of nine cases of previously healthy adults with prior EBV infection who experienced a rapid clinical course with fever, pancytopenia, and hepatic dysfunction; it was proposed that the condition seen in these cases be named progressive adult-onset EBV lymphoproliferative disorder, given their poor prognoses, although no clonal lymphocytes were identified.^6^ We hypothesize that these prior cases may involve EBV-HLH due to a sudden reactivation of latent EBV infection. While the precise mechanism underlying the sudden onset of HLH from latent infection remains unclear, it has been suggested that EBV latent membrane protein 1 (LMP-1) specifically inhibits the expression of signaling lymphocytic activation molecule-associated protein (SAP).^7^ This inhibition could potentially create an immunological deficiency similar to that observed in XLP1-associated primary HLH. These findings suggest that certain triggers in patients with latent EBV infection, even without underlying immunodeficiency, might induce an immunodeficient state reminiscent of that seen in primary HLH.^8^

The histology of the liver biopsy in the present case showed no evidence of malignant lymphoma but rather a proliferation of activated macrophages (epithelial histiocytes) accompanied by EBER-positive EBV-infected small lymphocytes. This histologic finding was consistent with the pathologic finding of EBV-HLH, in which EBV-infected lymphocytes activate macrophages and epithelial histiocytes replace normal tissue.^9^ It is not clear which lymphocyte subpopulation was infected with EBV; however, it is likely that EBV-infected lymphocytes were CD8-positive T cells, as the lymphocytes seen around the histiocytes were mostly T cells (specifically, predominantly CD8-positive T cells).

In adult-onset EBV-HLH, it is important to distinguish between non-neoplastic EBV-HLH and HLH secondary to EBV-related malignancy (primarily malignant lymphoma), as the prognosis and treatment strategies differ between these conditions.^2^ PET-CT scans are used to evaluate for the presence of malignancy, including malignant lymphoma. In non-neoplastic HLH, however, activated macrophages show high FDG uptake on PET-CT scans, similar to that seen in malignant lymphoma. Therefore, histologic examination is required for the accurate differential diagnosis of HLH. However, the rapid progression of EBV-HLH often results in insufficient time and/or medical conditions for obtaining a biopsy, and treatment must often be initiated on the basis of a clinical diagnosis. In the context of this challenging clinical diagnosis, it is important to recognize that EBV-HLH can suddenly develop from latent EBV infection; in the absence of this understanding, the diagnosis may be missed in some cases of EBV-HLH.

To date, there is no standard treatment for EBV-HLH in adults. The HLH-94/2004 treatment regimens, based on the protocols of international collaborative studies in HLH, consist of dexamethasone, cyclosporine, and etoposide and are most commonly used to suppress hyperinflammation, but relapse is often observed. For relapsed or refractory EBV-HLH, combination chemotherapy and hematopoietic stem cell transplantation are required.^1^^,^^2^ In the present case, the patient’s general condition rapidly deteriorated, with impaired consciousness two weeks after the onset of the disease; combination chemotherapy was selected after implementing pulse steroid therapy. As EBV-HLH is known to be caused by EBV-infected T cells or natural killer cells, we administered a reduced dose of SMILE therapy, which has demonstrated its effectiveness in natural killer/T-cell lymphoma, and achieved a successful outcome. Other cases involving successful SMILE therapy in EBV-HLH have also been reported, and this therapy can thus be considered a treatment option for EBV-HLH.^10^

## Conclusion

This case provides a significant clinical insight into EBV-HLH in adults. We demonstrated that EBV-HLH can develop from latent EBV infection even in immunocompetent adults without a history of CAEBV or EBV-associated malignancy. This finding expands our understanding of the pathogenesis of EBV-HLH and suggests the need for careful consideration of this diagnosis in adults presenting with HLH symptoms, regardless of their EBV-related medical history. This observation contributes to our understanding of the clinical spectrum of EBV-HLH and may help improve the early recognition of this life-threatening condition in adult patients.

## Informed consent

Informed consent was obtained from the patient for publication of this case report.

## Author contributions

MM and SU drafted the manuscript. All authors were involved in the diagnosis, treatment, and follow-up of the patient, critically revised the manuscript, read, and approved the current version of the manuscript.

## Conflicts of interest

The authors declare no conflicts of interest.
